# Global health collaborative research: beyond mandatory collaboration to mandatory authorship

**DOI:** 10.1186/s41256-023-00334-x

**Published:** 2023-11-22

**Authors:** Wongani John Nyangulu

**Affiliations:** Public Health and Nutrition Research Group, Department of Nutrition, Kamuzu University of Health Sciences, Blantyre, Malawi

**Keywords:** Questionable authorship practices, Power asymmetry in global health research, Authorship in global health research, Global health research partnerships

## Abstract

Collaborative research between the global north and global south is common and growing in number. Due to inability of local governments to fund research, global north actors provide the bulk of research funding. While providing mutual benefits, global collaborative research projects are far from ideal. In this paper, we review the authorship discrepancies in global collaborative research, discuss preventive measures in place and their shortfalls, and recommend an intervention to address the problem. Malawi research guidelines recommend collaboration between foreign and local researchers in locally conducted research. However, there is no provision requiring joint authorship in final published papers. Journal recommendations on authorship criteria exist, but they can disadvantage low- and middle-income country researchers in collaborative projects because of exclusionary interpretations of guidelines. For example, the requirement for authors to make substantial contributions to conception or design of the work may favor research grant holders, often from the global north. Systematic and holistic changes proposed to address power asymmetries at the core of the problem have been proposed. However, these proposals may take a long time to produce change. Ad interim, local institutions can take more direct action to address inequalities by establishing offices of research integrity to enforce mandates to increase opportunities for authorship in collaborative research.

## Background

Global research partnerships are common and often involve collaborative projects between funders and researchers from the global north, and researchers and communities from the global south [1]. These research partnerships have grown due to advances in technology and statistical methods for the analysis of large data sets which enable involvement of multiple, international research partners in local research projects. However, the primary limitation to growth has been African governments’ limited capacity and readiness to fund local research, leading to global north actors stepping in to provide research funding for local disease threats and to meet global academic interests [1].

The main feature of these collaborations are centers of partnership between global north and south institutions. The hosting countries benefit from these centers of partnership in many ways. They provide employment opportunities, and opportunities for career development through sponsored postgraduate training. They provide financial resources to fund research that addresses local health needs, producing innovations such as the Blantyre Coma Scale in Malawi [2]. Other benefits include sharing knowledge and expertise, and technology transfer allowing local institutions, and hospitals to access lifesaving technology. However, despite these benefits, these partnerships are far from ideal. In this paper, we review the authorship discrepancies in global collaborative research, discuss preventive measures in place and their shortfalls, and recommend an intervention to address the problem. 

## Questionable practices in global north–south research partnerships

There are several studies which highlight questionable authorship practices including one where 1593 articles on randomized controlled trials were evaluated for the period from 1990 to 2013 [3]. Among these articles, only half (49.8%) had low middle-income country (LMIC) first authors [3]. LMIC first and last authorship was lowest in Africa compared to the Americas and South East Asia [3]. LMIC first and last author representation was also lowest in research funded by high-income countries but highest in research funded by LMICs [3].

Another review study involving 786, 779 publications found that 86% had LMIC first authors [4]. However, the proportion of first authors was higher among the upper and lower-middle-income countries compared to low-income countries, mostly in Africa [4]. In African countries, first authorship was consistently below 50% [4]. Lastly, a systematic review focusing on collaborative research studies in Africa from 2013 to 2016 reviewed 7100 articles on health research. Over half (52.9%) had local first authors while 13.5% had no African co-authors at all even though they were done in Africa [5]. The lowest representation for African authors occurred when they worked with researchers from the USA, Canada, and Europe [5]. The highest representation was noted in the global south–south partnerships [5].

Underrepresentation of LMIC researchers in the prestigious first and last authorship positions negatively impacts career prospects as these matrices are used to evaluate success in academia, determine hiring and promotion to senior faculty positions and allocation of research grant funding.

## Local and international safeguards against questionable research practices

In Malawi, the National Commission for Science and Technology (NCST) was established to play an advisory role to Government in all matters related to science and technology. Concerning health research, the NCST has delegated oversight powers to the National Health Sciences Research Committee (NHSRC) and the College of Medicine Research Ethics Committee (COMREC). While the NHSRC is responsible for oversight over studies of national interest, COMREC has an oversight role over studies involving investigators affiliated with the University. The NCST and NHSRC also develop guidelines for health research in the country. These guidelines require that foreign researchers intending to do research in Malawi must be affiliated with local institutions and researchers who will participate in the research in a meaningful way and must have arrangements for local capacity building. However, these guidelines do not define what meaningful collaboration looks like especially concerning authorship criteria. There is no requirement regarding authorship in general and this represents a missed opportunity to enhance the representation of LMIC researchers in these roles.

Several international research guidelines specify authorship criteria for peer-reviewed journals. The most widely adopted are the International Committee of Medical Journal Editors (ICMJE) criteria [6]. Unfortunately, they have often been used to exclude researchers from LMICs because of exclusionary interpretation of the guidelines [6]. For example, the first criteria state an author must make “Substantial contributions to the conception or design of the work; or the acquisition, analysis or interpretation of data for the work” [6]. In collaborative research, HIC partners who are often the grant holders, are also responsible for the conception and design of the work [6, 7]. LMIC researchers are primarily responsible for the acquisition of data while analysis and interpretation often fall back on HIC researchers. The “or” in the statement suggests these roles are equally valued but as is often the case, data collection is often undervalued in the schema of research contributions [6, 7].

The second criteria state that an author should contribute to “Drafting the work or revising it critically for important intellectual content” [6]. English, being the most common publishing language may disadvantage LMIC researchers who are usually not native English speakers or whose writing style may not conform to the scientific style of literature required in these journals [6]. While there are ways to interpret the ICMJE criteria to be more inclusive in allocating authorship, it should be noted that inherent biases favoring researchers from the global north exist [7]. It should also be noted that these criteria were drafted by a committee of international journal editors who are mostly HIC researchers themselves and not representative of the international community of researchers [8]. These criteria are representative of their values which are often Anglocentric and pro-western.

## Addressing questionable authorship practices

There is a growing interest and movement toward restructuring global health, and greater representation of indigenous researchers and knowledge systems [9, 10]. A prominent target for reform is the system for research funding and awarding of research grants which favors HIC researchers and perpetuates power asymmetries in collaborative research projects [6]. Currently, there are calls to increase the allocation of funding to researchers in LMICs [6]. While this is welcome, the current mechanism for awarding grants which relies on excellence as an indicator of who should be funded also creates scenarios where research funding is concentrated in a few countries which have the capacity and established track record of research excellence including publications and authorship, about six of the 54 African countries [10]. It has been recommended that apart from excellence, equity should also be taken into account for external research funding programs to help lift countries that have not been competitive and spur those that have been competitive to greater capacity building [10].

African countries should take a leading role to fund local research and live up to spending 1% of gross domestic product (GDP) on research and development [5]. If this commitment were honored, it would increase local research capacity, reduce dependence on external research funding and help address the inequalities in global health research. However, most countries have not met this pledge [5]. For example, the NCST in Malawi provides research funding opportunities through the small grants schemes. However, the total budget available falls far short of 1% of GDP. The interventions proposed to restructure global health may take time to produce change. Until then, local research institutions need to step up to ensure fairness in collaborative research authorship.

We propose establishing offices of research integrity within the existing regulatory and oversight bodies and mandating them to promote equitable recognition of research partners in authorship as an act of research integrity and where necessary, investigate incidents of research misconduct, where authorship allocation does not recognize local researchers. Furthermore, these offices should be empowered to implement corrective and preventive measures such as developing guidelines to mandate that in collaborative research done in Malawi, the first and/or last authorship roles should be shared equitably among research partners depending on the preference or order of seniority in the research team and/or according to journal specification. This is important because while general research guidelines have been developed, none specifically relate to authorship. As part of the research approval process, this requirement could be an agreement made before study initiation and documented as part of the protocol submission to the research ethics committees to add further weight to the importance of such considerations.

There are potential challenges to implementing offices of research integrity including the need to recruit and train staff, the need for institutional buy-in from leadership and funding. However, one way of mitigating these challenges would be to utilize the same personnel involved in research ethics, approval and monitoring to take up the role of promoting research integrity. For example, compliance officers who conduct monitoring of research studies could also be trained to be research integrity officers. This would reduce costs related to hiring new staff and would encourage support from leadership.

## Conclusions

The lack of adequate representation in authorship is a hallmark of the current global health research system. While efforts to address this are implemented globally, local action to mandate more equitable authorship roles may be required. This could be the function of offices of research integrity established within the existing oversight institutions to promote more equitable authorship practices.

## Data Availability

Not applicable.

## Abbreviations

LMIC: Low middle-income country
LIC: Low income country
HIC: High income country
NCST: National Commission for Science and Technology
NHSRC: National Health Sciences Research Committee
COMREC: College of Medicine Research Ethics Committee
GDP: Gross domestic product
